# Liquid chromatography-tandem mass spectrometry assay for simultaneous quantification of catecholamines and metabolites in human plasma and cerebrospinal fluid

**DOI:** 10.1016/j.plabm.2025.e00471

**Published:** 2025-04-17

**Authors:** Yuting Wang, Quan Li, Yuhang Deng, Wenqing Wu, Cuiping Zhang, Yichi Zheng, Ming Guan, Haoqin Jiang

**Affiliations:** a Department of Laboratory Medicine, Huashan Hospital, Fudan University, Shanghai, 200040, China; b Huashan CSF Laboratory, Huashan Hospital, Fudan University, Shanghai, 200040, China

**Keywords:** Catecholamine, Metanephrine, Plasma, Cerebrospinal fluid, Liquid chromatography-tandem mass spectrometry

## Abstract

Catecholamines (CAs) and their metabolites in human cerebrospinal fluid (CSF) and plasma are potential biomarkers of Alzheimer's disease (AD) and facilitate early diagnosis. Liquid chromatography-tandem mass spectrometry is the gold standard method for analyzing CAs. The objective of this study was to develop and validate a liquid chromatography-tandem mass spectrometry assay capable of simultaneously quantifying dopamine (DA), epinephrine (E), norepinephrine (NE), metanephrine (MN), normetanephrine (NMN), and 3-methoxytyramine (3-MT) in both human CSF and plasma. Samples were processed by solid-phase extraction with a weak cation exchange adsorbent and then separated using an ultra-performance reversed-phase chromatography column. Analyte detection was performed using a triple quadrupole mass spectrometer operated in positive-ion multiple reaction monitoring mode. The developed assay was validated according to standard guidelines. The linearity, specificity, precision, accuracy, carryover and stability were assessed to ensure compliance with specified criteria. The lower limits of quantification for DA, E, NE, MN, NMN, and 3-MT were 4.5, 2.5, 4.5, 2.5, 2, and 0.3 pg mL^−1^, respectively. The total runtime for a single sample was 6.5 min. These results demonstrated that the method was sensitive, rapid, and reliable for the simultaneous quantification of DA, E, NE, MN, NMN, and 3-MT in clinical practice. We successfully detected CAs and their metabolites in plasma and CSF samples from patients with normal cognition and AD. This study demonstrates an efficient laboratory workflow for high-throughput analysis of CAs and their metabolites and lays a foundation for further studies on AD biomarkers.

## Highlights

• Six analytes (catecholamines and their metabolites) are analyzed simultaneously.
•Plasma and cerebrospinal fluid specimens are processed by weak cation exchange solid-phase extraction.
•he developed method does not require a derivatization step.
•Liquid chromatography-tandem mass spectrometry analysis takes only 6.5 min.

## 1 Introduction

Alzheimer's disease (AD) is the predominant cause of dementia in individuals aged over 65 years and is characterized by progressive impairment of cognitive function and behavior [1]. AD is officially listed as the fifth-leading cause of death in the United States [2]. The prevalence of AD is 3.21 % among people aged over 65 years, with over 7 million AD patients in China [3]. According to the most recent data, the global prevalence of dementia is projected to triple by 2050 [4]. Therefore, AD imposes a heavy social and economic burden on individuals, families, communities, and countries worldwide.

Studies have shown that AD patients exhibit either decreased or increased concentrations of various neurotransmitters in the brain, which are related to learning and memory [5]. Catecholamines (CAs) are endogenous compounds that function as neurotransmitters and hormones, which are pivotal in modulating responses to psychological and environmental stresses [6]. CAs, including epinephrine (E), norepinephrine (NE), and dopamine (DA), are primarily secreted by the adrenal medulla of the adrenal glands. Additionally, metanephrine (MN), normetanephrine (NMN), and 3-methoxytyramine (3-MT) are enzymatically produced through the action of catechol-O-methyltransferase on E, NE, and DA, respectively [7]. Numerous studies have indicated that CAs and their metabolites mirror the progress of AD and associated cognitive decline [5]. NE's functions relate to features of AD clinical presentation such as cognition, memory storage and retrieval, vigilance, and mood [8]. Additionally, a considerable body of evidence indicates that DA is involved in memory consolidation and spatial learning, and DA released from noradrenergic neurons in the locus coeruleus boosts memory retention [9]. A previous study indicated that E concentrations in cerebrospinal fluid (CSF) increase in patients with cognitive decline and in AD patients with severe dementia [10]. The AD patients had higher E and NE concentrations in CSF, but lower DA concentrations compared to the cognitively unimpaired [11]. NE concentration in CSF was significantly higher in the patients with advanced Alzheimer's disease than in those with mild to moderate severity, normal older subjects, or normal young subjects [12]. NE is significantly depleted in AD patients and that along with its depletion there is down regulation of many metabolites within the dopamine pathway [13]. However, the results for the relationship between AD and E in plasma samples are inconsistent [14]. Moreover, little research has been conducted on the concentrations of catecholamine final metabolites in Alzheimer's disease. Therefore, to further explore the association between CAs and AD, there is an urgent need to establish a sensitive, specific, and reliable method for detecting CAs and their metabolites.

Numerous analytical methods have been developed for quantitative analysis of CAs including immunoassays [15], radioimmunoassays [16], high-performance liquid chromatography (HPLC) [17], and mass spectrometry [18]. The advantages and disadvantages of these four methods are shown in Table S1. Immunoassays used to detect hormones are based on antigen–antibody reactions. However, cross-reactions between antigens and antibodies result in interference and overestimation [19]. Radioimmunoassays require manual processing, which is time-consuming and accompanied by isotope hazards, while chromatographic analysis often requires laborious sample preparation procedures, including derivatization [18]. To overcome these limitations, liquid chromatography-tandem mass spectrometry (LC-MS/MS) has been developed, and it is now recognized as the gold standard for analyzing CAs. LC-MS/MS provides significant advantages in the determination of CAs, including for the sensitivity, specificity, precision, and speed of the analysis. Multiple studies have investigated different techniques for sample preparation for the detection of CAs by LC-MS/MS. However, some methods only permit the detection of individual CAs rather than the simultaneous detection of multiple CAs, which results in the procedures being time-consuming and expensive.

CSF is a colorless fluid located in the subarachnoid spaces and ventricles of the brain that promotes the normal development and functioning of neural cells in the brain. CSF is the biofluid located closest to the brain, and the CSF composition directly reflects alterations in the pathophysiology of the central nervous system. Although CSF is collected using invasive techniques, its diagnostic value for neurological diseases far exceeds that of plasma and urine, especially in AD [20,21].

Studies on the detection of CAs in urine [7,22] or plasma [23] are relatively abundant, whereas those on the detection of CAs in CSF are scarce because of the low availability of samples. Research on CA detection in CSF is currently limited, and few studies have analyzed CAs in both CSF and plasma or investigated the correlations between these two types of samples.

In this study, we focused on establishing a method for the simultaneous detection of CAs and their metabolites in CSF and plasma. The goal was to establish a robust and sensitive mass spectrometry method for correlation studies on plasma and CSF in patients with AD. We developed a sensitive LC-MS/MS assay for measuring CAs (DA, E, and NE) and their metabolites (MN, NMN, and 3-MT) in plasma and CSF samples simultaneously. By eliminating the need for a derivatization step, we developed a rapid method that enabled the simultaneous detection of six different substances in one injection. The validated method was successfully applied to quantify CAs and their metabolites in plasma and CSF samples from selected patients with normal cognition (NC) or AD in a clinical laboratory.

## 2 Materials and methods

### 2.1 Clinical specimens

Plasma and CSF samples were collected from patients after routine testing at Huashan Hospital (Shanghai, China). All patients or guardians of the patients participated voluntarily and provided their informed consent at the time of admission to the hospital for medical examinations. The protocol was reviewed and approved by the Ethics Committee of Huashan Hospital (KY2023-505). Plasma samples were obtained from a cohort of 10 individuals clinically diagnosed with AD and an equal number of age-matched NC individuals as controls. Moreover, CSF specimens were collected from eight patients with AD and an equivalent number of NC individuals. The plasma and CSF groups were two independent experimental groups and not paired with each other. AD patients were diagnosed according to the core clinical criteria released in 2011 by the National Institute on Aging and Alzheimer's Association (NIA-AA 2011) [24]. CSF samples from healthy individuals were unavailable. The NC patients had no cognitive impairments but were diagnosed with brain tumors at the Neurosurgery Department of Huashan Hospital. All samples were stored at −80 °C before analysis.

### 2.2 Reagents and instruments

A triple quadrupole liquid chromatography-mass spectrometry system (ARP6465MD, Rongjia Biotech Co., Ltd., Shanghai, China) was used to separate and quantify DA, E, NE, MN, NMN, and 3-MT. Standard compounds (DA, E, NE, MN, NMN, and 3-MT) and isotopically labeled internal standards (ISs) (DA-*d*_4_, E-*d*_6_, NE-*d*_3_, MN-*d*_3_, NMN-*d*_3_, 3-MT-*d*_4_) were obtained from CMASS (Shanghai, China). The isotopic purities of the ISs are shown in Table S2. Zero-point calibration was performed with the ISs but no spiked analytes (Figures Sl and S2). Hormone-free plasma and phosphate buffered saline were purchased from SeraCare (Milford, MA, USA) and Solarbio (Beijing, China), respectively. Artificial CSF (sterilized) was purchased from Coolaber (Beijing, China). The hormone-free plasma and artificial CSF did not contain endogenous molecules (Figs. S3 and S4). Formic acid (HPLC grade), ammonium acetate (HPLC grade), and acetic acid (LC-MS/MS grade) were purchased from Aladdin (Shanghai, China). Acetonitrile and methanol (LC-MS/MS grade) were purchased from Merck KGaA (Darmstadt, Germany). OASIS WCX 96-well plates 30 μm (30 mg) and ACQUITY 96-well collection plates were obtained from Waters Corp. (Milford, MA, USA).

### 2.3 Stock and working solutions

Acetic acid (10 mL) was added to 1000 mL of deionized water to obtain 1 % aqueous acetic acid. Standard stock solutions of DA, E, NE, MN, NMN, and 3-MT (1 mg/mL) were prepared using 1 % aqueous acetic acid. Mixed stock solutions A and B containing DA, E, NE, MN, NMN, and 3-MT were also prepared using 1 % aqueous acetic acid. The concentrations of DA, E, NE, MN, NMN, and 3-MT in mixed stock solution A were 72, 40, 72, 40, 32, and 4.8 ng/mL, respectively. The concentrations of DA, E, NE, MN, NMN, and 3-MT in mixed stock solution B were 9, 30, 45, 11, 10, and 2 ng/mL, respectively.

Mixed stock solution A was gradually diluted with hormone-free plasma and CSF to obtain working standard samples. Mixed stock solution B was also diluted with hormone-free plasma and CSF to obtain low-value quality control (LQC), middle-value quality control (MQC), and high-value quality control (HQC) solutions (Table S3). DA-*d*_4_, E-*d*_6_, NE-*d*_3_, MN-*d*_3_, NMN-*d*_3_, and 3-MT-*d*_4_ were separately mixed with 1 % aqueous acetic acid and then diluted with more 1 % aqueous acetic acid to obtain IS working solutions with concentrations of 3, 3, 3, 1, 5, and 3 ng/mL, respectively.

### 2.4 Sample preparation and solid-phase extraction

Blood (2 mL of whole blood) and CSF (2 mL) samples were drawn from participants in the morning. Blood was collected into K2 EDTA plasma tubes and centrifuged at 4000 rpm for 10 min at 4 °C. The resulting plasma was subsequently aliquoted into 1.5-mL polypropylene tubes and stored at −80 °C. CSF was obtained by lumbar puncture and centrifuged at 950 rpm for 10 min at 4 °C, and the supernatant was then stored in polypropylene tubes at −80 °C.

Activated eluent (acetonitrile, 200 μL) was first added to a 96-well solid-phase extraction (SPE) plate. After leaving the columns to dry, they were equilibrated by adding 200 μL of 0.2 mmol/L ammonium acetate. The CSF and blood samples (500 μL) were vortex mixed with 50 μL of IS solution and 500 μL of buffer solution (0.2 mmol/L ammonium acetate). Then, 1000 μL of the mixture was transferred to the activated SPE plate. The flow rate was regulated using a positive pressure instrument. Ammonium acetate (200 μL, 0.2 mmol/L) was added for leaching. After standing for 5 min, the plate was gradually pressed dry with a positive pressure device. Formic acid (5 % in methanol, 200 μL) was added for elution, and again after standing for 5 min, the plate was pressed dry with the positive pressure device. The elution operation was repeated twice, and the eluent was collected into a round bottom 96-well plate. The collected eluent was dried under a stream of nitrogen gas at 30 °C, and then 60 μL of 0.1 % formic acid in water was added to redissolve the samples. After centrifugation, the supernatant was retained for measurement.

### 2.5 LC-MS/MS procedure

Separation was performed on a Kinetex F5 column (100 × 3 mm, 2.6 μm; Phenomenex, Torrance, CA, USA) with a total run time of 6.5 min at 40 °C. The mobile phase was a mixture of 0.1 % formic acid in water (mobile phase A) and methanol (mobile phase B). The gradient elution and flow rate are shown in Table S4. The last 0.4 min of elution was used for equilibration.

The quantitation and detection of DA, E, NE, MN, NMN, and 3-MT were performed using the triple quadrupole liquid chromatography-mass spectrometry system in positive electrospray ionization with the gas temperature, gas flow rate, and nebulizer pressure set at 180 °C, 5 L/min, and 45 psi, respectively. The capillary voltage, sheath gas temperature, and sheath gas flow rate were optimized at 2500 V, 350 °C, and 11 L/min, respectively. The quadrupole resolution was one atomic mass unit. The dwell time for each transition was 20 ms for DA, E, NE, MN, NMN, and 3-MT. Multiple reaction monitoring transitions were acquired for each of CAs and their isotopically labeled ISs in positive ionization mode. The mass-to-charge ratios and collision energies for the quantifier and qualifier ion transitions are summarized in Table S5. MassHunter Data Acquisition software (version 1.2.0) was used for data acquisition.

### 2.6 Method validation

The method was validated according to the Clinical and Laboratory Standards Institute C62-A standard [25] and Chinese guidance for clinical application of LC and mass spectrometry [26]. The linearity, lower limit of quantification (LLOQ), specificity, matrix effects, accuracy, precision, carryover and stability were all evaluated according to the guidelines.

#### 2.6.1 Linearity

Six point calibration curves were constructed using samples of hormone-free plasma and artificial CSF, with three replicates for each sample. Multiple regression analysis and linear equations were used to evaluate the linearity. The calibration curves were obtained by plotting the analyte-to-IS peak area ratios against the nominal concentrations of the ISs. Linearity was considered acceptable if the coefficient of determination (*R*^2^) was >0.99.

Working standard sample 6 prepared using plasma and CSF was diluted 10-, 5-, and 3-fold with 1 % low-fatty acid bovine serum albumin. Five replicates were prepared for each sample. The dilution consistency was evaluated and considered acceptable if the recovery was between 85 % and 115 % and the coefficient of variation (CV) was within ±15 %.

#### 2.6.2 LLOQ

The LLOQ of the method was defined as the lowest concentration that could be accurately determined. The LLOQs of all analytes met the requirements for accuracy and precision if the deviations between the detected and theoretical values were within 15 % and the CVs were within 20 %. All the samples were analyzed within the same analytical run.

#### 2.6.3 Specificity and matrix effect

The specificity was evaluated by analyzing five double-blank samples without the IS or analytes, five samples spiked at the LLOQ concentration, and five samples containing the ISs. The ratio of background peak area to the LLOQ or the ISs peak area was calculated. The specificity was validated when the background peak area/LLOQ peak area was <5 % and the background peak area/IS peak area was <1 %.

The matrix effect was estimated by comparing the peak responses of the post-extraction matrix of spiked plasma or CSF with those of standards in neat solvent at the three quality control concentrations. The matrix effect was calculated as a percentage using the following the equation: Matrix effect (%) = (*A*_1_ × *M*_2_)/(*A*_2_ × *M*_1_) × 100, where *A*_1_ is the peak area of the IS in the sample, *A*_2_ is the peak area of the IS in the standard solution, *M*_1_ is the mass of the IS in the sample, and *M*_2_ is the mass of the IS in the standard solution. The matrix effect at each concentration was determined five times, and ratios falling within the range of 80 %–120 % after IS correction were considered acceptable [27].

#### 2.6.4 Precision

Intra-assay precision was evaluated using LQC, MQC, and HQC solutions prepared from the CSF and plasma samples. The intraday precision was determined by repeating the experiments five times per day, and the interday precision was determined by repeating the experiments five times over three different days. Both the intra- and inter-assay precision met the requirements if the CVs were within ±15 %.

#### 2.6.5 Recovery

To evaluate the accuracy of the assay, a recovery experiment was performed. Pure ISs were added to the CSF and plasma samples, and these samples were subsequently analyzed six times. The recovery was calculated as a percentage using the following the equation: Recovery (%) = (*C*_1_ − *C*_2_)/*C*_0_ × 100, where *C*_1_ is the calculated concentration in the spiked sample, *C*_2_ is the calculated concentration in the non-spiked sample, and *C*_0_ is the theoretical concentration of the analyte added to the sample.

#### 2.6.6 Carryover and stability

The carryover was evaluated by injecting a blank sample immediately after injection of a high concentration sample. Working standard samples with higher concentration were selected, and the concentrations of DA, E, NE, MN, NMN and 3-MT were 3600, 2000, 3600, 2000, 1600, 240 pg mL^−1^, respectively. The carryover peak area in the subsequent blank sample injection should not exceed 20 % of the LLOQ and 5 % of the IS.

The stability of the analytes in the samples and calibration solutions was assessed by analyzing the calibration, CSF, and plasma quality control samples under different handling and storage conditions. LQC, MQC, and HQC samples were stored at room temperature for 2 h, −20 °C for 3 days, and −80 °C for 7 days and then analyzed five times. The method was considered acceptable if the deviation of the mean concentration was within 15 % at each level.

## 3 Results and discussion

### 3.1 Method development

#### 3.1.1 Sample preparation

The mobile phase, SPE eluent, pH, and reaction time were optimized for the pretreatment. There is currently no uniform, mature, or standard pretreatment method for CSF. Because the CSF matrix is simple, we attempted to use the dilution method from a previous study for sample pretreatment [11], but the standard curve was not linear (Table S6). Therefore, we investigated using SPE to process the CSF and plasma samples.

SPE is performed by retaining target compound(s) on a solid-phase adsorbent and then eluting the analyte(s) with an organic solvent. The distribution of the analyte(s) in the two phases is influenced by the pH. The stationary phase in a weak cation exchange SPE column contains weak cation exchange groups, which carry positive charges under certain pH conditions. In the present study, this type of column gave high extraction efficiencies for CAs and its metabolites, and satisfactory linearity and recovery.

Our method, which eliminates the need for derivatization, offers a more streamlined and time-efficient workflow compared to the derivatization method for CSF reported by Peng et al. [28]. This is particularly important for clinical applications where rapid and reliable results are crucial. Moreover, our method successfully detects catecholamines and their metabolites in both plasma and CSF samples, providing a comprehensive assessment of these biomarkers in different biological matrices.

#### 3.1.2 Chromatography and mass spectrometry

CAs and their metabolites are more stable under acidic conditions than under neutral or basic conditions. In our experiments, the polar functional groups of the Kinetex F5 column allowed for the CAs and their metabolites to be stabilized under the mobile phase conditions [0.1 % formic acid in water (v/v) and methanol and eluted rapidly. This column is particularly suitable for retaining and separating polar compounds.

In this study, 5 % formic acid in methanol was used as the eluent and the pH was adjusted to 2. The mild acidic environment increased the effectiveness of CAs elution.

**Fig. 1:**
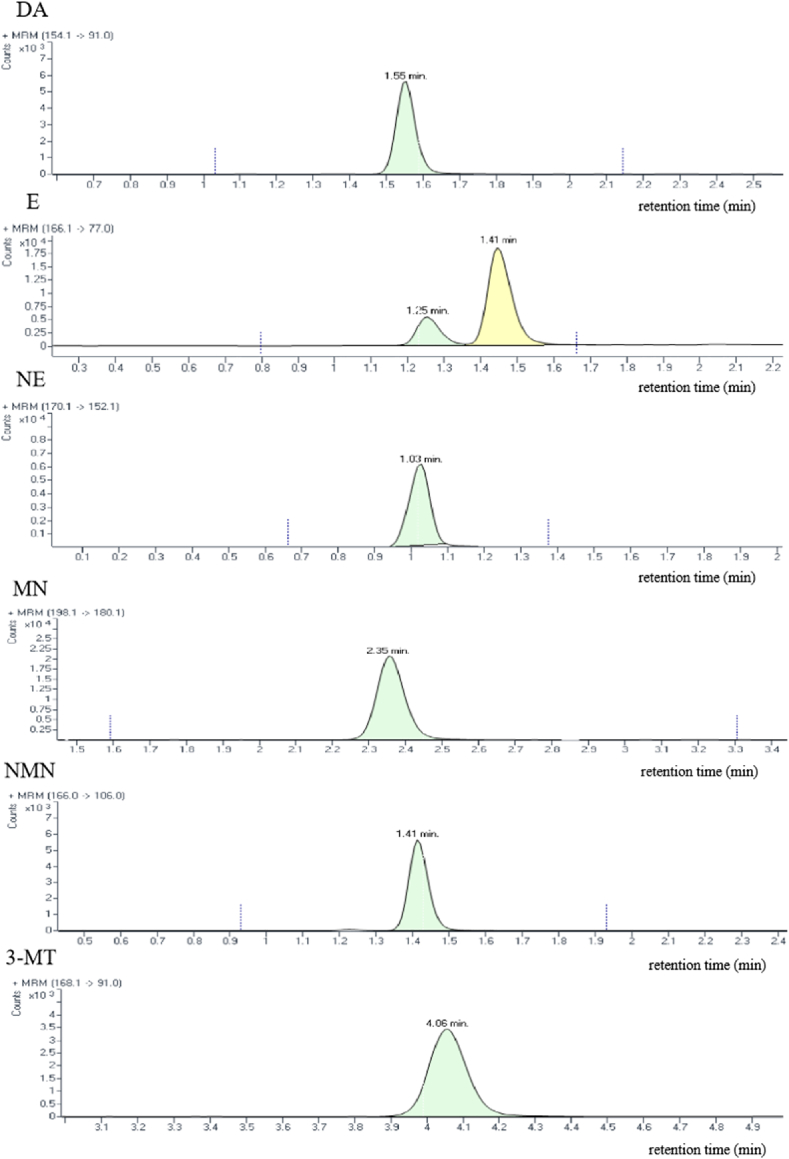
Representative chromatograms showing signals of standard dopamine (DA), epinephrine (E), norepinephrine (NE), metanephrine (MN), normetanephrine (NMN), and 3-methoxytyramine (3-MT). The concentrations of DA, E, NE, MN, NMN, and 3-MT were 900, 500, 900, 500, 400 and 60 pg mL^−1^, respectively.

The total runtime for standard substances was 6.5 min, with a post time of 1.5 min. The retention times of DA, E, NE, MN, NMN, and 3-MT were 1.55, 1.25, 1.03, 2.35, 1.41, and 4.06 min, respectively (Fig. 1). E and NMN are isomers with a molecular weight of 183.20 and a dehydrated molecular weight of 166. In our results, the peak at 1.25 min was for E and that at 1.41 min was for NMN. Catecholaminergic molecules contain amine groups and these amine groups readily accept protons to form stable positive ions in mass spectrometry. This means that they can be detected in positive electrospray ionization mode. Mass spectrometry detection and quantification of all compounds and their isotope internal standards were performed using Data Acquisition software and Quant-My-Way software. In contrast to the study conducted by Yu et al. [23], our method can be completed by adapting to HPLC, the lower LLOQ of DA was also obtained. The advantages of our method in sensitivity and sample type make it more valuable in practical applications, especially in scenarios where high sensitivity detection is required.

Overall, the method developed in this study improved the extraction efficiency, simplified the pretreatment, reduced the analysis time, adjusted the peak shape, reduced tailing, and gave acceptable, reliable, and highly sensitive results.

### 3.2 Method validation

#### 3.2.1 Linearity

To analyze human plasma and CSF samples containing DA, E, NE, MN, NMN, and 3-MT at concentrations over a broad range, six calibration curves were established and validated for linearity. The calibration samples contained DA and NE at concentrations of 4.5–3600 pg mL^−1^, E and MN at concentrations of 2.5–2000 pg mL^−1^, NMN at concentrations of 2–1600 pg mL^−1^, and 3-MT at concentrations of 0.3–240 pg mL^−1^ in plasma and CSF. All curves were linear with *R*^2^ > 0.99 (Table 1). The dilution consistency was evaluated by diluting sample solutions 10-, 5-, and 3-fold. The results were acceptable with recoveries of 85 %–115 % and CVs within ±15 % (Table S7).

**Table 1:** Calibration range and lower limit of quantification (LLOQ) of each analyte.

Analyte	Calibration range (pg mL^−1^)	R^2^	R^2^	LLOQ (pg mL^−1^) (n = 5)		CV (%) (n = 5)	
		plasma	CSF	plasma	CSF	plasma	CSF
DA	4.5–3600	0.9989	0.9958	4.5	4.5	10.3	6.1
E	2.5–2000	0.9993	0.9953	2.5	2.5	10.3	8.9
NE	4.5–3600	0.9984	0.9999	4.5	4.5	8.9	4.8
MN	2.5–2000	0.9998	0.9977	2.5	2.5	2.0	6.9
NMN	2–1600	0.9987	0.9936	2	2	2.0	10.9
3-MT	0.3–240	0.9985	0.9972	0.3	0.3	3.7	5.2

Abbreviations: DA, dopamine; E, epinephrine; NE, norepinephrine; MN, metanephrine; NMN, normetanephrine; 3-MT, 3-methoxytyramine; CV, coefficient of variation, and CSF, cerebrospinal fluid. *R*^2^ is the coefficient of determination.

#### 3.2.2 LLOQs

The LLOQs of DA, E, NE, MN, NMN, and 3-MT in the plasma and CSF samples were 4.5, 2.5, 4.5, 2.5, 2, and 0.3 pg mL^−1^, respectively. The CVs of all analytes ranged from 2.0 % to 10.9 % (Table 1). Because of the low concentration of CAs in CSF, a sample volume of 500 μL was needed to obtain a good instrument response. The sensitivity was not negatively affected by small volumes of plasma and CSF samples.

#### 3.2.3 Specificity and matrix effect

For hormone-free plasma samples, the ratios of the background peak area/LLOQ peak area and the background peak area/IS peak area of the six analytes were within 3.80 % and 0.026 %, respectively. Similarly, the ratios of the background peak area/LLOQ peak area and the background peak area/IS peak area of the six analytes in the artificial CSF samples were within 4.27 % and 0.026 %, respectively. These results showed that the specificity of this method was acceptable (Table 2).

**Table 2:** Specificity and matrix effect for plasma and cerebrospinal fluid (CSF) samples.

	Analyte	Specificity (n = 5)		Matrix effect (n = 5)			
		Background peak area/LLOQ peak area (%)	Background peak area/IS peak area (%)	LQC (%)	MQC (%)	HQC (%)	Range (%)
Plasma samples	DA	0.16	0.026	101.3	98.0	100.6	94.3–107.1
	E	3.80	0.021	98.5	94.9	96.7	85.9–113.4
	NE	2.33	0.003	107.3	103.9	98.3	94.6–110.2
	MN	1.37	0.012	100.9	100.8	98.7	96.3–104.0
	NMN	1.33	0.019	100.7	99.5	101.6	91.0–109.9
	3-MT	1.00	0.024	98.3	101.4	98.7	92.2–109.3
CSF samples	DA	0.16	0.026	99.6	99.3	100.3	97.4–103.3
	E	4.27	0.021	100.7	96.0	99.0	85.7–110.3
	NE	2.46	0.002	107.3	102.4	99.5	96.1–111.0
	MN	1.29	0.012	99.6	99.3	100.3	89.5–108.1
	NMN	1.27	0.019	96.6	99.3	99.2	90.8–103.5
	3-MT	2.15	0.023	100.0	97.8	100.7	93.4–106.2

The ratio of the background peak area to the LLOQ or IS peak area (*n* = 5) was used to evaluate the specificity. The matrix effect was determined (*n* = 5) at LQC, MQC and HQC concentrations.

Abbreviations: DA, dopamine; E, epinephrine; NE, norepinephrine; MN, metanephrine; NMN, normetanephrine; 3-MT, 3-methoxytyramine; LLOQ, lower limit of quantification; IS, internal standard; LQC, low-value quality control; MQC, middle-value quality control; and HQC, high-value quality control. *R*^2^ is the coefficient of determination.

After correction with isotopically labeled ISs, the ratios were all between 80 % and 120 %. For plasma, the minimum was 85.92 % and maximum was 113.43 %, and for CSF, the minimum was 85.71 % and maximum was 110.98 %. These results showed that the matrix effects were acceptable.

#### 3.2.4 Precision and accuracy

Both the intra- and inter-assay precision met the requirement that CVs should be within ±15 %. The highest CV values at the LLOQs of all the analytes were 10.9 % and 11.6 % for the plasma and CSF samples, respectively (Table 3, Table 4). The mean extraction recovery rate ranges at the LLOQ were 86.0 %–98.2 % for DA, 90.3 %–110.2 % for E, 89.5 %–100.5 % for NE, 93.7 %–109.1 % for MN, 87.8 %–111.9 % for NMN, and 99 %–110.4 % for 3-MT in the plasma samples. For the CSF samples, the mean extraction recovery rates at the LLOQ were 85.4 %–108.6 % for DA, 80.9 %–109.5 % for E, 82.8 %–119.5 % for NE, 90.2 % to 119.4 for MN, 81.7 %–110.5 % for NMN, and 100.1 %–118.6 % for 3-MT. Therefore, the repeatability of this method was good. The accuracy was evaluated using recovery experiments with the LQC, MQC, and HQC samples. In the LQC, MQC, and HQC plasma samples, the mean extraction recovery rate ranges were 94.3 %–104.30 % for DA, 90.1 %–101.4 % for E, 95.1 %–104.2 % for NE, 95.8 %–104.4 % for MN, 100.7 %–103.2 % for NMN, and 94.8 %–102.5 % for 3-MT. For the LQC, MQC, and HQC CSF samples, the mean extraction recovery rate ranges were 95.7 %–109.9 % for DA, 93.5 %–106.5 % for E, 97 %–111.8 % for NE, 99 %–109.4 % for MN, 98.1 %–108.4 % for NMN, and 101.7 %–110.6 % for 3-MT. Therefore, the accuracy of the method was acceptable because the recovery rates for the LQC, MQC, and HQC samples were between 85 % and 115 %, and the recovery rates at the LLOQ varied from 80 % to 120 %.

**Table 3:** Precision and accuracy for analysis of plasma samples.

Analyte		Theoretical concentration (pg mL^−1^)	Intra-assay (n = 5)		Inter-assay (n = 15)		Recovery (n = 5)
			Mean (pg mL^−1^)	CV (%)	Mean (pg mL^−1^)	CV (%)	Mean (range, %)
DA	LLOQ	4.5	4.12	6.1	4.36	9.7	91.6 (86.0–98.2)
	LQC	90	85.21	2.7	94.36	8.5	94.3 (91.7–97.2)
	MQC	450	455.55	2.8	446.78	5.0	101.2 (96.9–104.6)
	HQC	2880	3005.56	0.5	2942.34	2.5	104.3 (103.9–105.3)
E	LLOQ	2.5	2.49	8.9	2.45	9.2	99.6 (90.3–110.2)
	LQC	50	45.07	2.2	49.26	7.8	90.1 (87.3–92.2)
	MQC	250	249.87	2.8	244.96	5.2	99.9 (96.9–103.7)
	HQC	1600	1622.19	2.5	1620.99	3.6	101.4 (98.1–103.9)
NE	LLOQ	4.5	4.21	4.8	4.47	8.7	93.6 (89.5–100.5)
	LQC	90	96.1	1.9	103.94	8.0	95.1 (93.0–97.8)
	MQC	450	479.34	1.8	447.9	6.6	104.2 (101.3–106.4)
	HQC	2880	2983.23	2.7	2830.18	5.7	103.2 (98.9–106.1)
MN	LLOQ	2.5	2.56	6.9	2.56	5.7	102.3 (93.7–109.1)
	LQC	50	57.52	1.4	58.47	6.2	95.8 (93.2–97.3)
	MQC	250	270.70	2.0	260.87	5.1	104.4 (102.0–107.6)
	HQC	1600	1621.02	2.5	1613.50	2.6	100.7 (96.9–103.8)
NMN	LLOQ	2	1.99	10.9	1.89	10.0	99.7 (87.8–111.9)
	LQC	40	49.48	1.7	52.25	6.6	100.7 (97.7–102.9)
	MQC	200	215.63	3.7	207.05	4.7	103.2 (99.0–109.7)
	HQC	1280	1312.22	3.3	1281.75	3.8	101.8 (98.4–106.7)
3-MT	LLOQ	0.3	0.32	5.2	0.30	8.8	105.3 (99–110.4)
	LQC	6	5.89	2.6	6.22	7.3	94.8 (91.6–97.5)
	MQC	30	30.96	2.4	30.28	4.0	102.5 (99–105)
	HQC	190	195.85	1.2	192.31	3.2	101.9 (100.4–103.5)

The mean and CV of intra-assay (*n* = 5) and inter-assay (*n* = 15) analyses were used to evaluate the precision. The mean and range of recovery (*n* = 5) at four analyte concentrations were used to evaluate the accuracy.

Abbreviations: DA, dopamine; E, epinephrine; NE, norepinephrine; MN, metanephrine; NMN, normetanephrine; 3-MT, 3-methoxytyramine; CV, coefficient of variation; LLOQ, lower limit of quantification; LQC, low-value quality control; MQC, middle-value quality control; and HQC, high-value quality control.

**Table 4:** Precision and accuracy for the analysis of cerebrospinal fluid (CSF) samples.

Analyte		Theoretical concentration (pg mL^−1^)	Intra-assay (n = 5)		Inter-assay (n = 15)		Recovery (n = 5)
			Mean (pg mL^−1^)	CV (%)	Mean (pg mL^−1^)	CV (%)	Mean (range, %)
DA	LLOQ	4.5	4.23	4.9	4.28	10.2	94.5 (85.4–108.6)
	LQC	90	87.77	1.4	89.80	2.9	95.7 (94.7–98.0)
	MQC	450	496.21	1.1	481.49	3.7	109.9 (108.3–111.4)
	HQC	2880	2983.85	3.2	2949.16	2.8	103.6 (99.4–108.1)
E	LLOQ	2.5	2.29	11.6	2.66	2.8	99.1 (80.9–109.5)
	LQC	50	46.77	5.6	49.66	5.8	93.5 (86.9–101.1)
	MQC	250	266.23	2.5	259.77	6.6	106.5 (102.6–109.0)
	HQC	1600	1643.66	1.0	1623.84	3.3	102.7 (101.4–103.8)
NE	LLOQ	4.5	4.92	7.2	4.34	11.5	102.8 (82.8–119.5)
	LQC	90	98.73	1.7	99.88	3.7	97.0 (96.8–100.7)
	MQC	450	514.46	1.7	478.75	6.2	111.8 (108.9–113.7)
	HQC	2880	3008.22	1.4	2880.10	7.5	104.1 (101.6–105.4)
MN	LLOQ	2.5	2.86	3.2	2.50	9.4	107.2 (90.2–119.4)
	LQC	50	56.22	3.7	56.92	3.6	99.0 (95.4–106.1)
	MQC	250	280.19	1.4	274.15	3.4	109.4 (107.4–111.5)
	HQC	1600	1681.21	1.9	1663.25	1.7	104.7 (102.3–106.7)
NMN	LLOQ	2	2.04	5.3	1.75	6.6	94.9 (81.7–110.5)
	LQC	40	43.78	2.4	44.83	4.3	98.1 (94.3–101.5)
	MQC	200	221.36	3.1	215.82	5.9	108.4 (103–111.9)
	HQC	1280	1329.02	3.1	1298.05	3.4	103.5 (99.4–108.0)
3-MT	LLOQ	0.3	0.31	2.2	0.34	5.2	107.6 (100.1–118.6)
	LQC	6	6.50	5.1	6.5	5.2	101.7 (93.4–106.9)
	MQC	30	33.57	2.4	32.12	6.5	110.6 (106.8–114.0)
	HQC	190	200.00	2.3	195.75	4.2	104.0 (100.2–106.0)

The mean and coefficient of variation (CV) of intra-assay (*n* = 5) and inter-assay (*n* = 15) analyses were used to evaluate the precision. The mean and range of recovery (*n* = 5) at four analyte concentrations were used to evaluate the accuracy.

Abbreviations: DA, dopamine; E, epinephrine; NE, norepinephrine; MN, metanephrine; NMN, normetanephrine; 3-MT, 3-methoxytyramine; LLOQ, lower limit of quantification; LQC, low-value quality control; MQC, middle-value quality control; and HQC, high-value quality control.

To further ensure the clinical applicability of the plasma assay, our laboratory successfully participated in the National Center for Clinical Laboratories (NCCL) Proficiency Testing Program (Project ID: NCCL-C-38) for CAs and metabolites. All target analytes (DA、E、NE、MN、NMN、3-MT) met the NCCL acceptance criteria, confirming the accuracy of our method in plasma matrix.

#### 3.2.5 Carryover and stability

After the injection of a high concentration sample, the concentration of six analytes in the subsequent blank sample was calculated. The analysis results indicated that the carryover concentrations for all six analytes were all below the LLOQ, thereby meeting the acceptance criteria.

The mean recovery rates were between 85 % and 115 % of the initial value for all six analytes in the plasma and CSF samples at the three concentrations (LQC, MQC, and HQC) after storage at room temperature for 2 h, −20 °C for 3 days, and −80 °C for 7 days. These results indicated that the stability was acceptable (Tables S8 and S9). Therefore, routine sample collection procedures will not affect the results for the detection of CAs and metabolites and the developed LC-MS/MS method is suitable for clinical practice.

### 3.3 Clinical application

The developed method was applied to analyze non-paired experimental groups of plasma and CSF. The plasma cohort comprised 10 NC patients and 10 AD patients, while the CSF cohort consisted of eight NC patients and eight AD patients. The NC patients were diagnosed with brain tumors but had no cognitive impairments. Notably, DA, E, NE, MN, NMN, and 3-MT in plasma and CSF from NC and AD patients were successfully detected using the LC-MS/MS method (Table 5). Representative chromatograms showing the signals for DA, E, NE, MN, NMN, and 3-MT in plasma are provided in Figs. S5–S8.

**Table 5:** Mean concentrations of catecholamines (pg mL^−1^) in plasma and cerebrospinal fluid (CSF) from normal cognition (NC) patients and Alzheimer's disease (AD) patients and comparison with published data.

Patient characteristics	Plasma Samples				CSF Samples			
	Reference [23]	Reference [29]	Our method		Reference [28]	Reference [33]	Our method	
	Healthy Adults	Healthy Adults	NC	AD	Viral and Bacterial meningitis	Healthy Volunteers	NC	AD
	N = 73	N = 23	N = 10	N = 10	N = 200	N = 35	N = 8	N = 8
Age	21–68	42.0 ± 8.7	62.9 ± 7.3	60.8 ± 6.4	0.5–2	≥48	57.8 ± 12.8	61.1 ± 9.2
Women	51/71	13/23	7/10	6/10	/	/	6/8	3/8
Analytes								
DA	10.20 ± 4.60	20.40 ± 3.53	38.53 ± 25.33	7.09 ± 2.24	15.32 ± 10.72	13.79 ± 15.23	35.13 ± 26.55	17.16 ± 7.39
E	29.30 ± 14.20	34.79 ± 4.72	17.59 ± 10.07	27.87 ± 26.15	5.50 ± 5.50	/	2.24 ± 1.05	6.53 ± 7.8
NE	427.00 ± 190.60	/	2325 ± 763.37	5418.73 ± 3352.04	118.43 ± 60.90	165.80 ± 15.23	159.62 ± 69.73	279.21 ± 76.8
MN	22.90 ± 7.20	/	15.1 ± 8.19	27.05 ± 15.17	17.83 ± 13.87	/	3.04 ± 1.89	2.56 ± 1.2
NMN	41.40 ± 17.20	/	59.62 ± 48.42	86.35 ± 64.54	131.90 ± 60.40	/	81.91 ± 33.8	112.34 ± 63.74
3-MT	2.34 ± 2.01	/	2.92 ± 2.11	0.19 ± 0.26	35.11 ± 20.07	/	5.48 ± 2.3	7.27 ± 4.68

Abbreviations: NC, normal cognition; DA, dopamine; E, epinephrine; NE, norepinephrine; MN, metanephrine; NMN, normetanephrine; and 3-MT, 3-methoxytyramine.

In both the NC and AD groups, the concentrations of DA, E, MN, NMN, and 3-MT in the plasma samples were generally consistent with those from previous research using LC-MS/MS [23,29]. Only the plasma NE concentrations differed significantly between our experimental groups and the previous research. For instance, plasma NE concentrations in the NC group and AD group were 2325 ± 763.37 pg mL^−1^ and 5418.73 ± 3352.04 pg mL^−1^, respectively, while those in a previous study were 427.00 ± 190.60 pg mL^−1^ [23]. The plasma NE concentrations were significantly higher in our experimental group than in the previous study. This difference might arise from the older age of participants in our experimental group (62.9 ± 7.3 years) than in the previous study (21–68 years) [23]. Numerous studies have shown that plasma NE concentrations increase with age, which might arise from a compensatory reaction induced by the diminished sensitivity of postsynaptic adrenergic receptors to NE [[30], [31], [32]]. For example, the average NE concentration is 66 % higher and the NE plasma clearance rate is 22 % lower in elderly individuals than in young people [32]. The concentrations of DA, E, NE, MN, NMN, and 3-MT in CSF samples in the present study were similar to those in previous studies [28,33]. Our results showed that the developed method effectively detected CAs and their metabolites in both plasma and CSF.

CAs can serve as neuromodulators in the central nervous system, and the loss of catecholaminergic regulation contributes to AD [5,34]. Prior research has demonstrated that AD patients exhibit increased NE concentrations and decreased DA concentrations. For instance, the plasma NE concentrations in AD patients with mini-mental state examination scores of >24 are significantly higher than those in non-AD patients [17]. Additionally, a recent study reported that AD patients (*n* = 294) had higher concentrations of CSF NE and lower concentrations of CSF DA than cognitively unimpaired people (*n* = 113) [11].

The performance of the developed method was validated and confirmed with a limited number of clinical specimens, and was generally consistent with previous studies [23,28,29,33]. We intend to further expand this research to more thoroughly investigate the relationship between CAs and AD.

## 4 Conclusions

A LC-MS/MS method was developed and validated for simultaneous quantification of CAs (DA, E, NE) and their metabolites (MN, NMN, and 3-MT) in plasma and CSF that was pretreated using SPE with a weak cation exchange adsorbent. The entire assay took only 6.5 min. We successfully detected CAs and their metabolites in plasma and CSF samples from patients with normal cognition and AD. This sensitive, rapid, and reliable method improves the efficiency of laboratory workflows and is feasible for the analysis of CSF. Our results provide a technological approach for future studies on biomarkers in AD patients.

## CRediT authorship contribution statement

**Yuting Wang:** Writing – original draft, Visualization, Methodology, Investigation, Formal analysis, Data curation, Conceptualization. **Quan Li:** Writing – original draft, Visualization, Methodology, Investigation, Formal analysis, Data curation. **Yuhang Deng:** Writing – review & editing, Supervision, Resources. **Wenqing Wu:** Supervision, Resources, Methodology. **Cuiping Zhang:** Supervision, Methodology. **Yichi Zheng:** Investigation. **Ming Guan:** Writing – review & editing, Supervision, Funding acquisition. **Haoqin Jiang:** Writing – review & editing, Supervision, Funding acquisition.

## Funding sources

This work was supported by the innovative medical device application demonstration project of the 10.13039/501100008838Shanghai Municipal Commission of Economy and Information, China (Grant No. 23SHS06200).

## Declaration of competing interest

The authors declare the following financial interests/personal relationships which may be considered as potential competing interests:Ming Guan reports financial support was provided by Innovative medical device application demonstration project of Shanghai Municipal Commission of Economy and Information. Haoqin Jiang reports financial support was provided by Innovative medical device application demonstration project of Shanghai Municipal Commission of Economy and Information. If there are other authors, they declare that they have no known competing financial interests or personal relationships that could have appeared to influence the work reported in this paper.

## Data Availability

Data will be made available on request.
