# Supplementary material for: Liquid chromatography-tandem mass spectrometry assay for simultaneous quantification of catecholamines and metabolites in human plasma and cerebrospinal fluid
Source: Pract Lab Med. 2025 Apr 17;45:e00471. doi: 10.1016/j.plabm.2025.e00471 (PMC12049989; doi:10.1016/j.plabm.2025.e00471)
Supplement: Multimedia component 1 [file mmc1.docx]

**Supplementary Materials**

Figure S1. Representative chromatograms showing signals of the internal standards (ISs) for dopamine (DA), epinephrine (E), norepinephrine (NE), metanephrine (MN), normetanephrine (NMN), and 3-methoxytyramine (3-MT) in plasma. The concentrations of DA-*d*_4_, E-*d*_6_, NE-*d*_3_, MN-*d*_3_, NMN-*d*_3_, and 3-MT-*d*_4_ were 3, 3, 3, 1, 5, and 3 pg/mL, respectively.


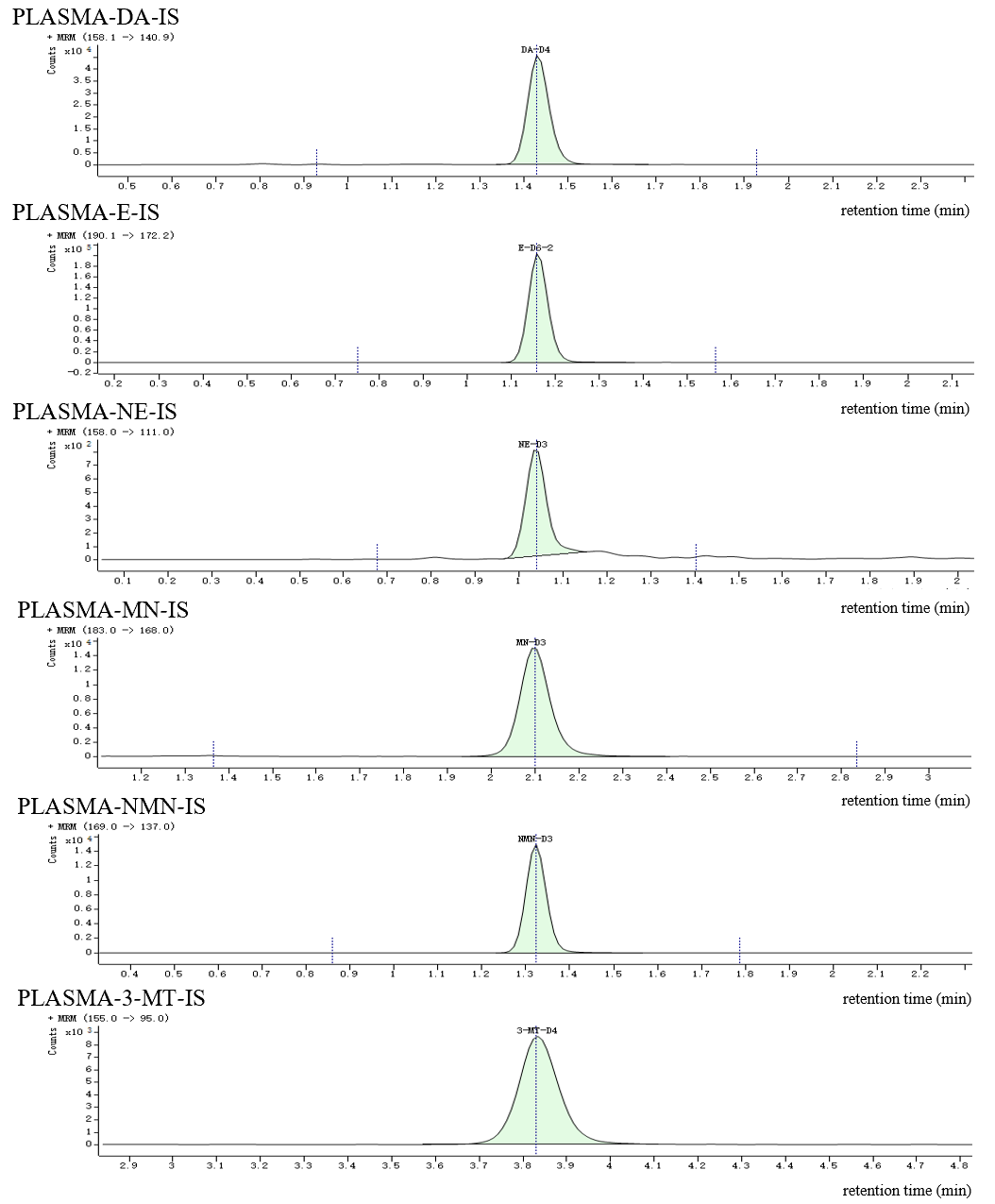


Figure S2. Representative chromatograms showing signals of the ISs for DA, E, NE, MN, NMN, and 3-MT in CSF. The concentrations of DA-*d*_4_, E-*d*_6_, NE-*d*_3_, MN-*d*_3_, NMN-*d*_3_, and 3-MT-*d*_4_ were 3, 3, 3, 1, 5, and 3 pg/mL, respectively.

**
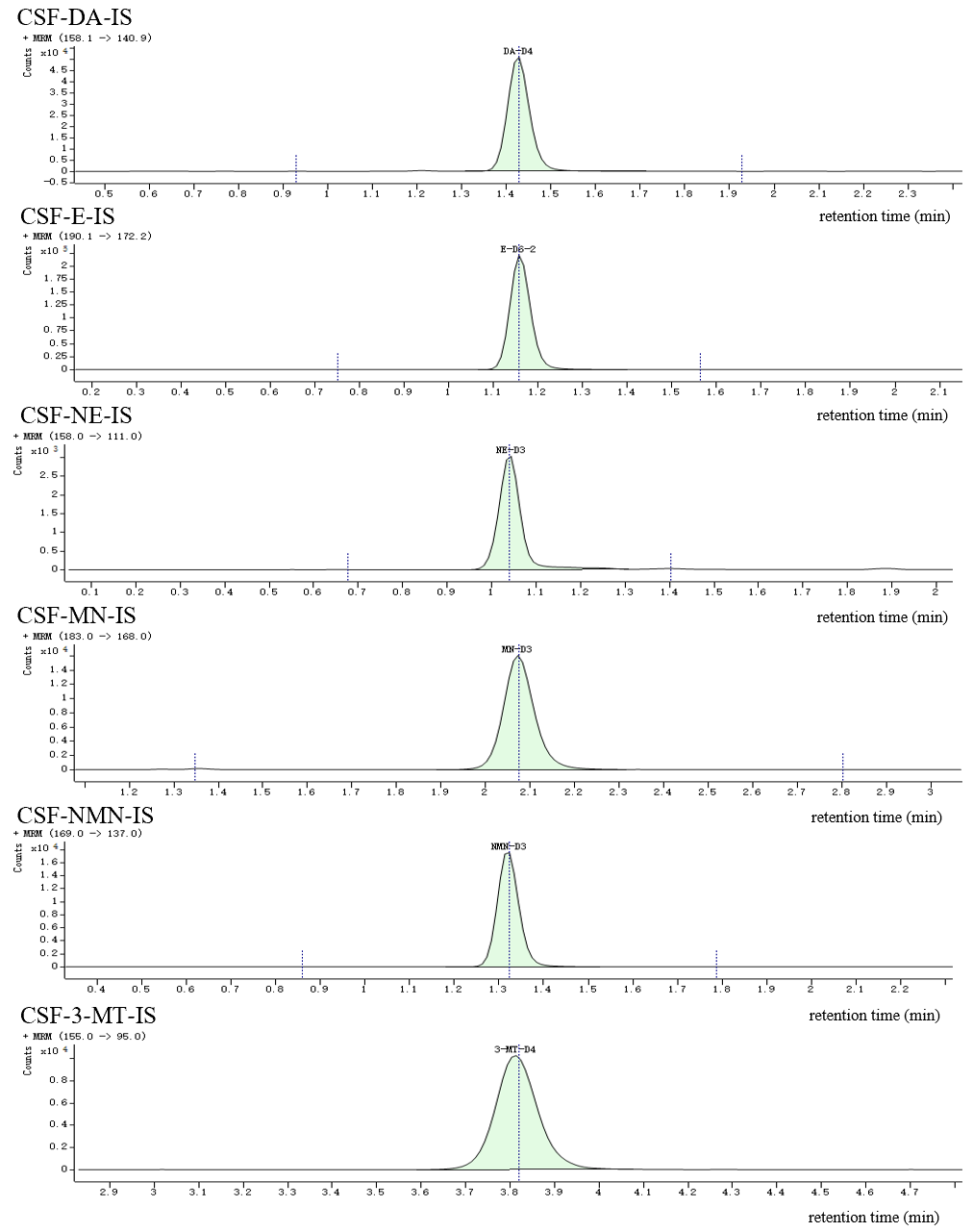
**

Figure S3. Representative chromatograms showing signals of hormone-free plasma and the LLOQs for DA, E, NE, MN, NMN, and 3-MT. The blank sample was hormone-free plasma without ISs or analytes. The LLOQs of DA, E, NE, MN, NMN, and 3-MT in the plasma samples were 4.5, 2.5, 4.5, 2.5, 2, and 0.3 pg/mL, respectively.


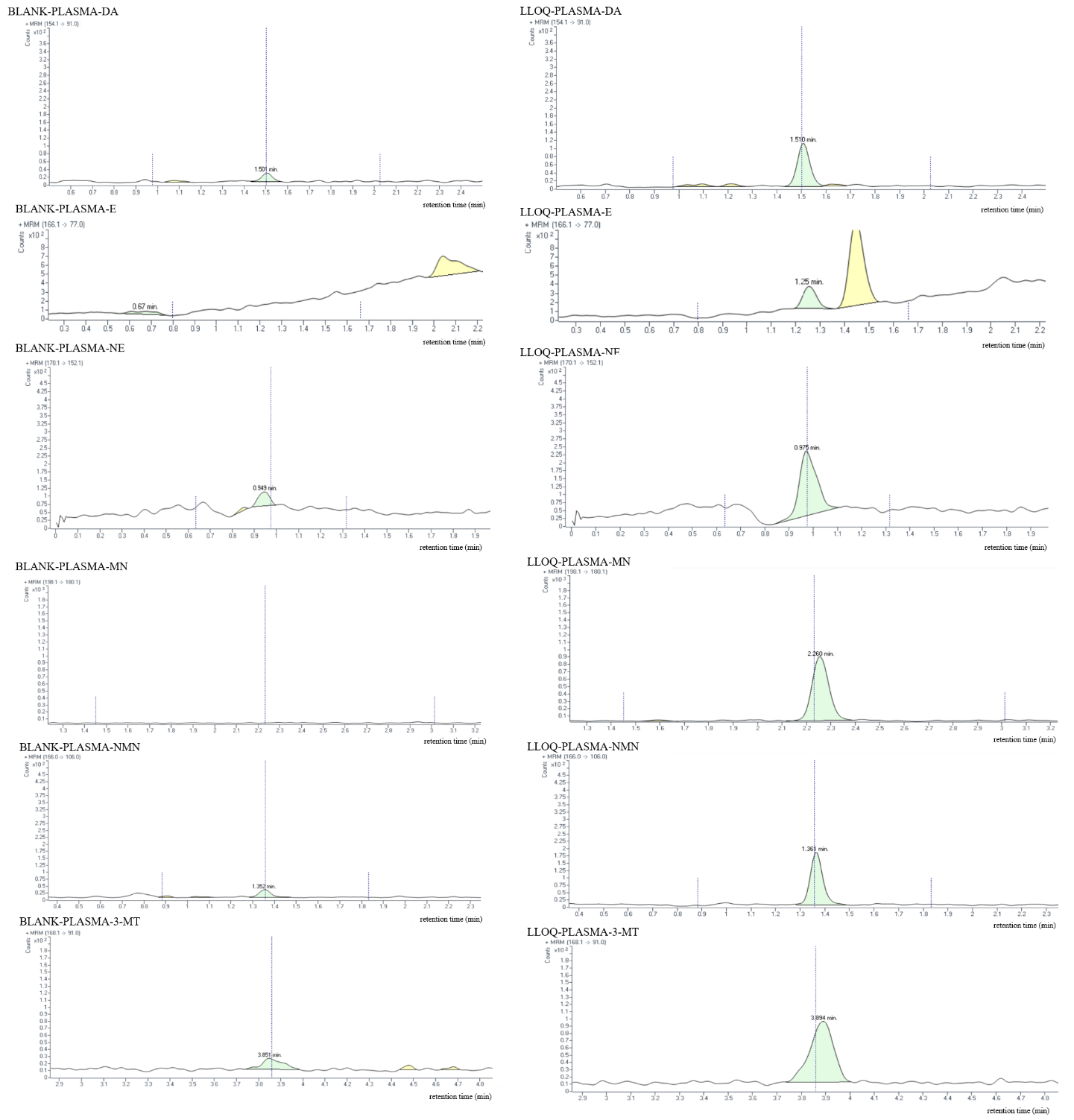


Figure S4. Representative chromatograms showing signals of hormone-free CSF and the LLOQs for DA, E, NE, MN, NMN and 3-MT. The blank sample was hormone-free CSF without ISs or analytes. The LLOQs of DA, E, NE, MN, NMN, and 3-MT in the CSF samples were 4.5, 2.5, 4.5, 2.5, 2, and 0.3 pg/mL, respectively.

**
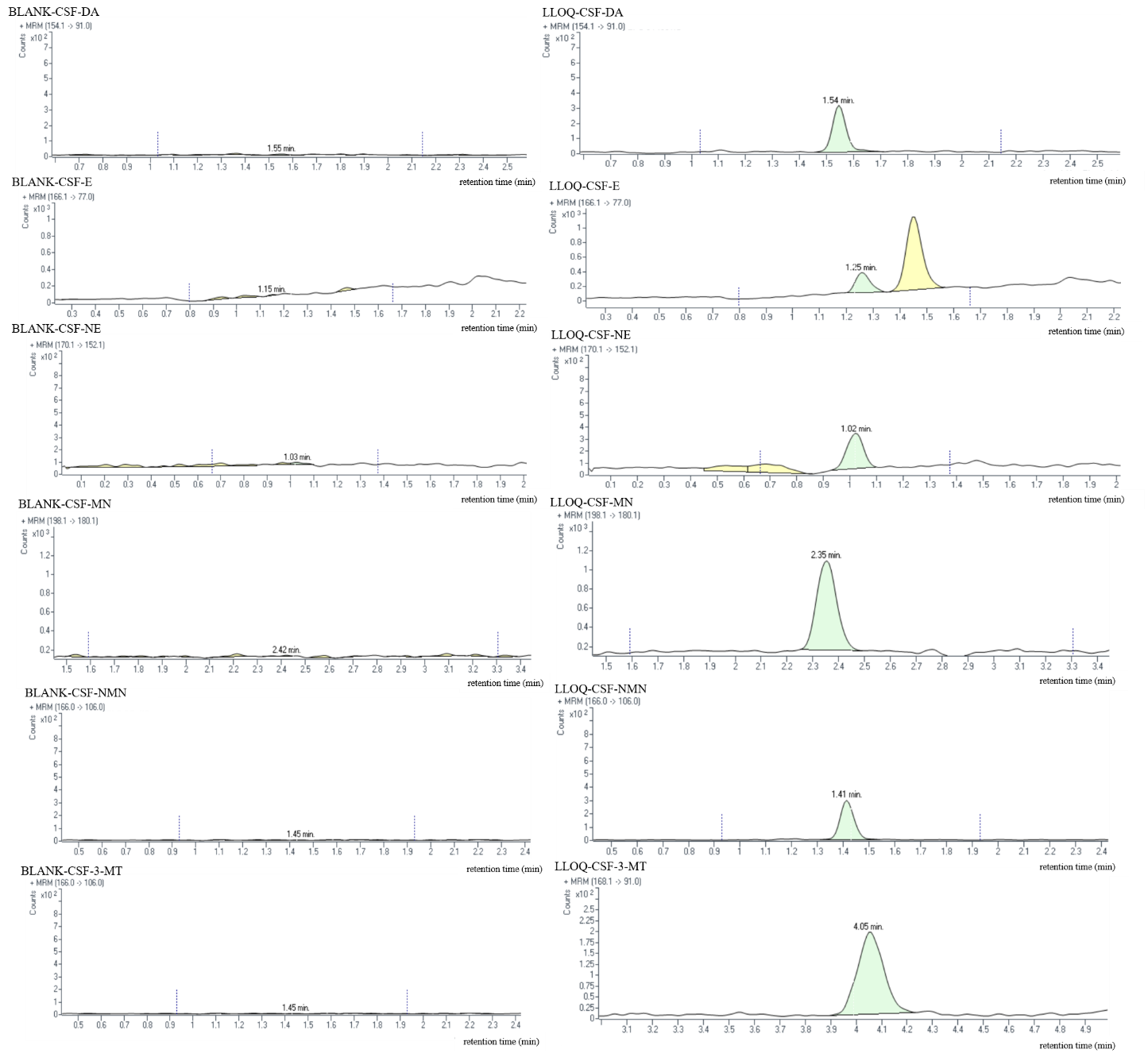
**

Figure S5. Representative chromatograms showing signals of DA, E, NE, MN, NMN, and 3-MT in plasma from a normal cognition (NC) patient. The concentrations of DA, E, NE, MN, NMN, and 3-MT in the plasma samples were 25.48, 42.61, 3049.65, 10.29, 40.02, and 0.27 pg/mL, respectively. The figures on the right (DA-IS, E-IS, NE-IS, MN-IS, NMN-IS, and 3-MT-IS) show the six internal standards in the plasma from the NC patient.


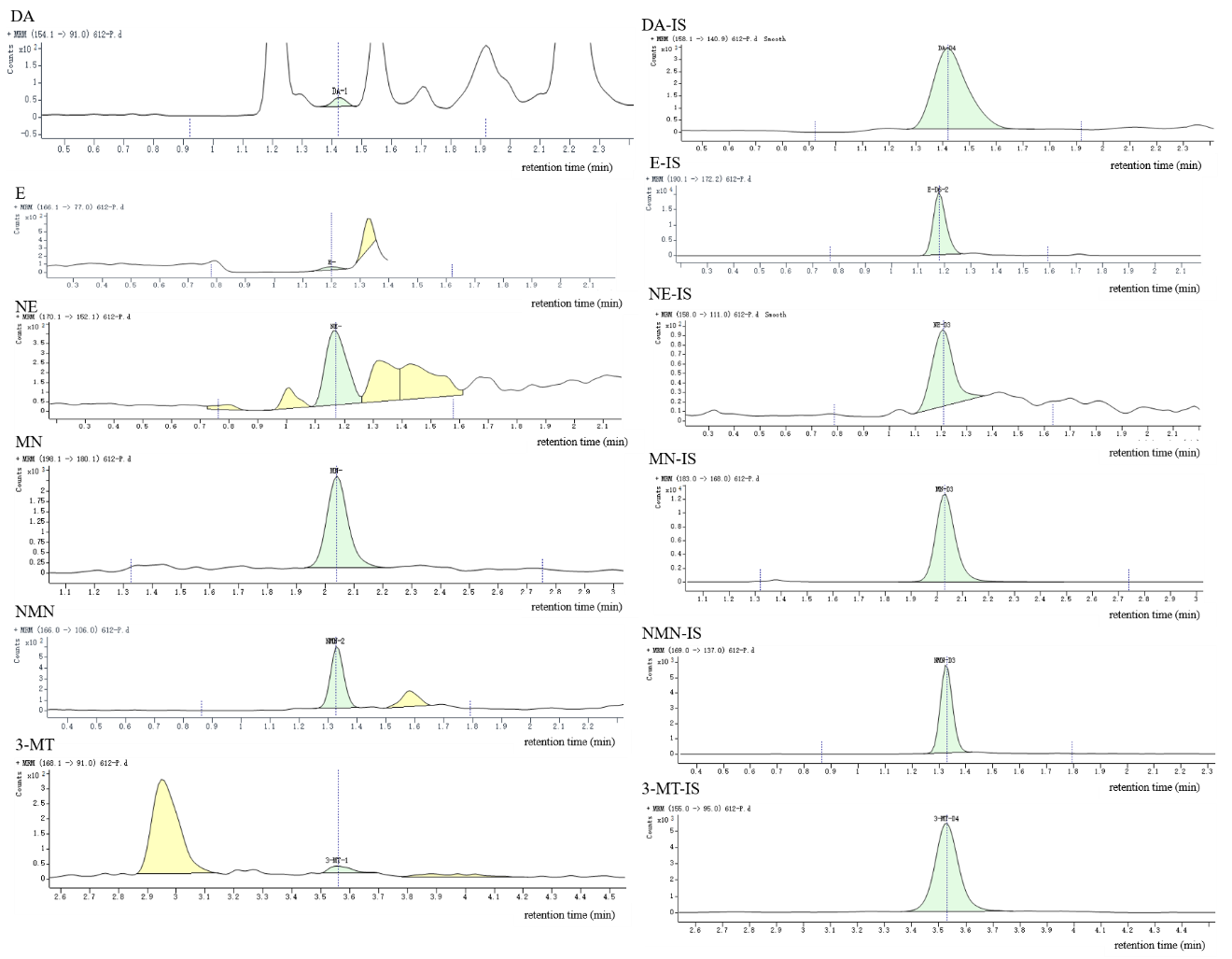


Figure S6. Representative chromatograms showing signals of DA, E, NE, MN, NMN, and 3-MT in CSF from a normal cognition (NC) patient. The concentrations of DA, E, NE, MN, NMN and 3-MT in the CSF samples were 15.89, 1.62, 222.74, 1.28, 128.12 and 7.46 pg/mL, respectively. The figures on the right (DA-IS, E-IS, NE-IS, MN-IS, NMN-IS, and 3-MT-IS) show the six internal standards in the CSF from the NC patient.


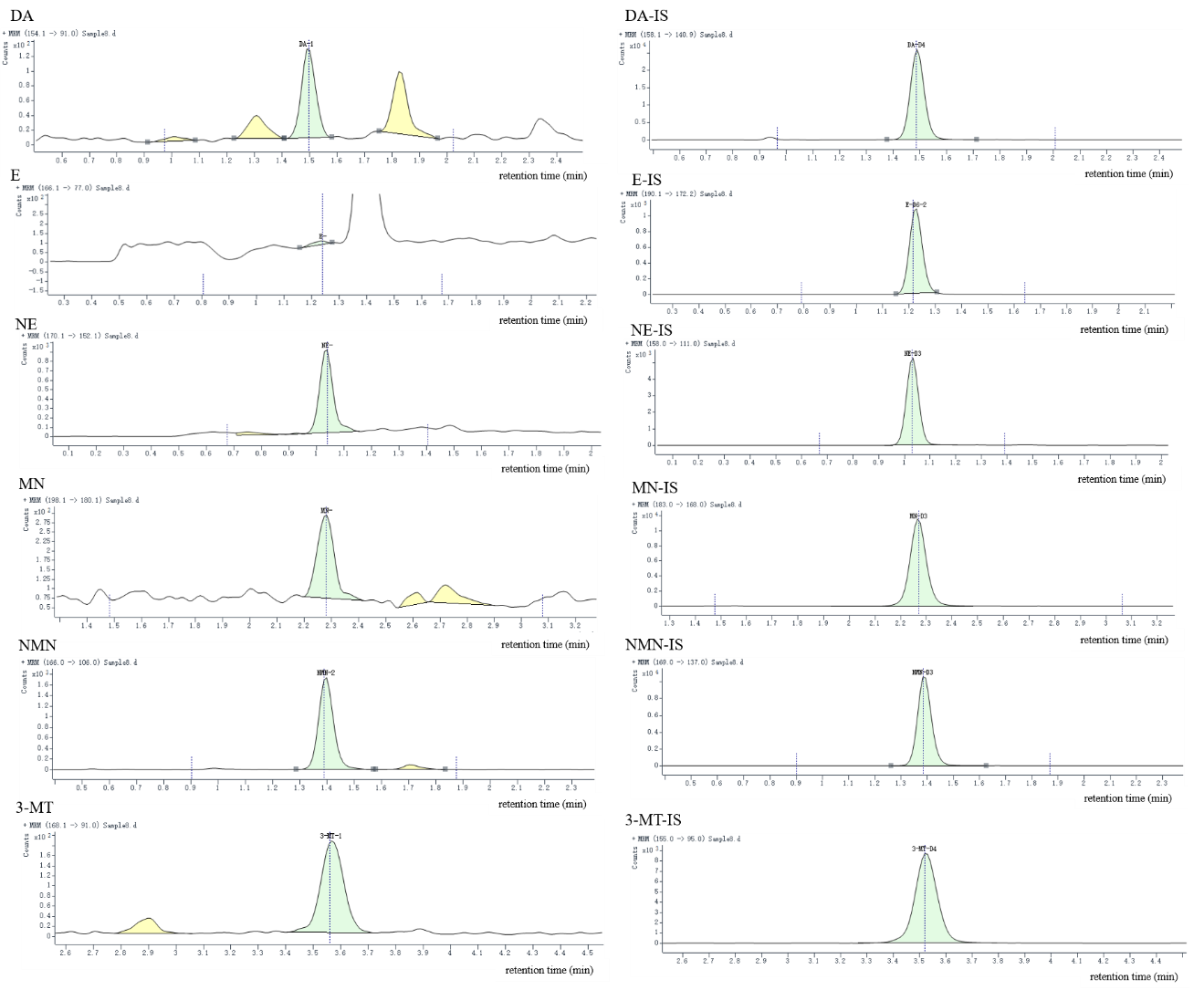


Figure S7. Representative chromatograms showing signals of DA, E, NE, MN, NMN, and 3-MT in plasma from a patient with AD. The concentrations of DA, E, NE, MN, NMN and 3-MT in the plasma sample were 5.58, 5.04, 1935.84, 4.07, 22.37 and 0.19 pg/mL, respectively. The figures on the right (DA-IS, E-IS, NE-IS, MN-IS, NMN-IS, and 3-MT-IS) shows the six internal standards in the plasma from the AD patient.


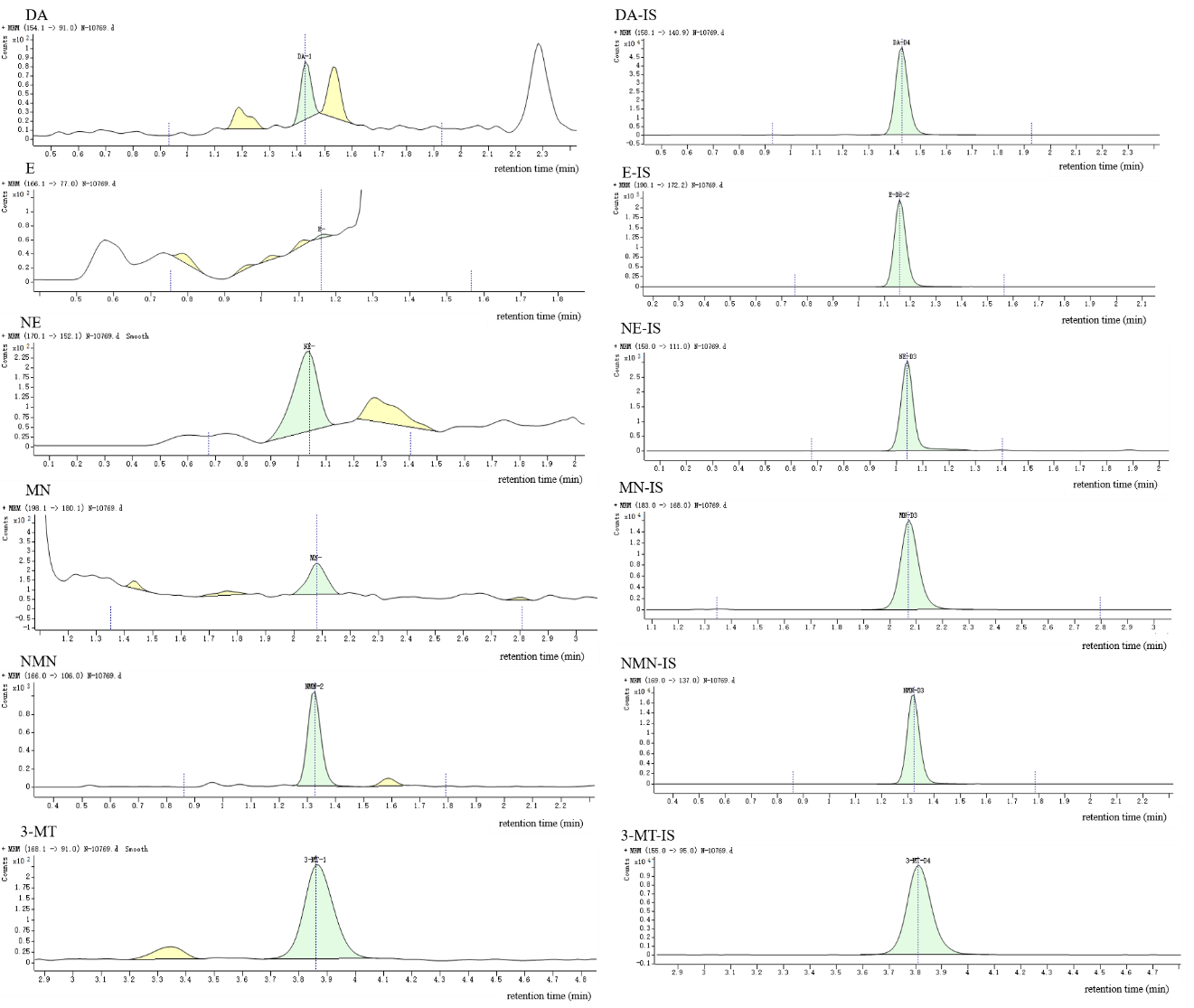


Figure S8. Representative chromatograms showing signals of DA, E, NE, MN, NMN, and 3-MT in CSF from a patient with AD. The concentrations of DA, E, NE, MN, NMN, and 3-MT in the CSF samples were 18.02, 0.13, 333.90, 5.13, 135.88, and 11.03 pg/mL, respectively. The figures on the right (DA-IS, E-IS, NE-IS, MN-IS, NMN-IS, and 3-MT-IS) show the six internal standards in the CSF from the AD patient.


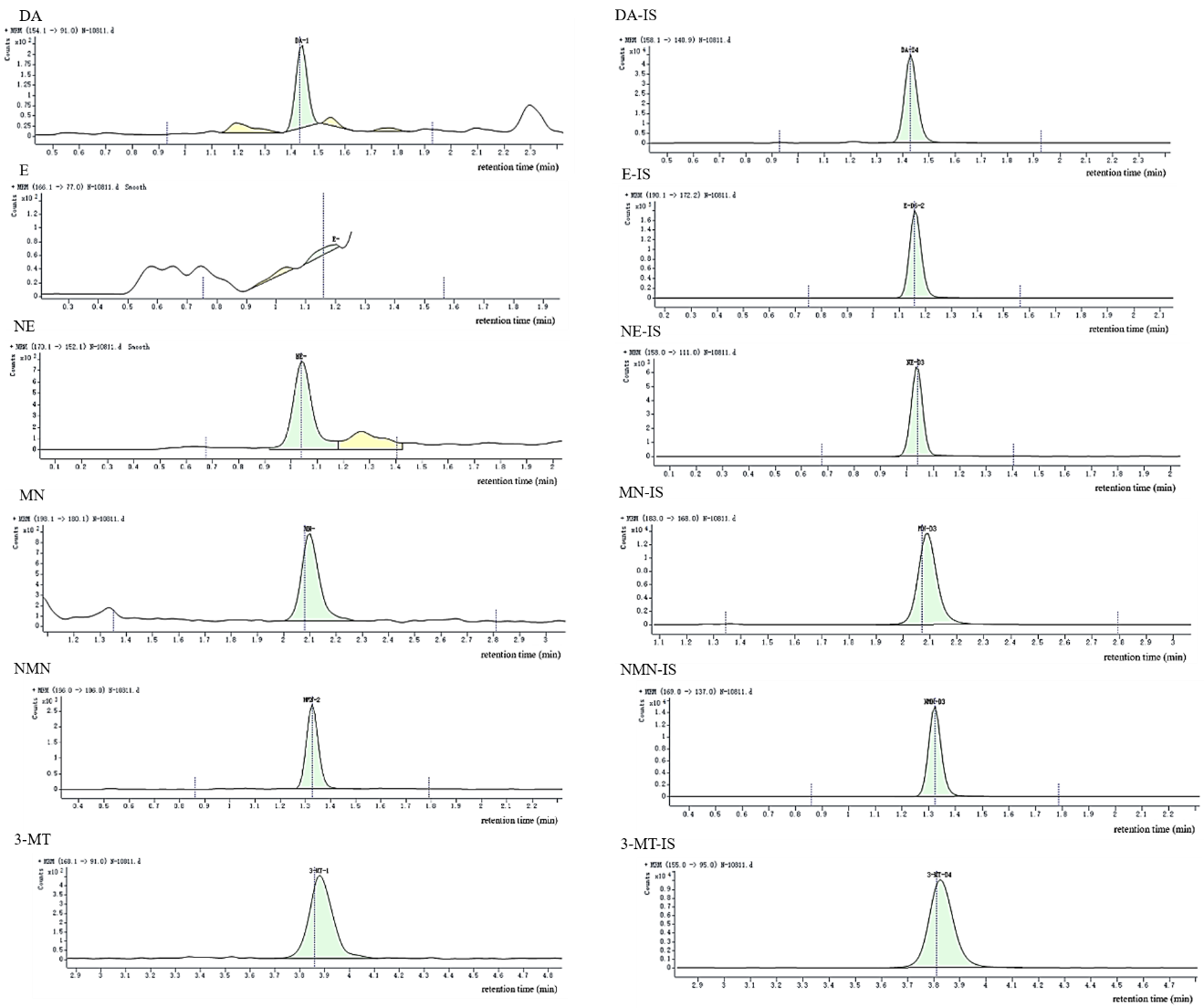

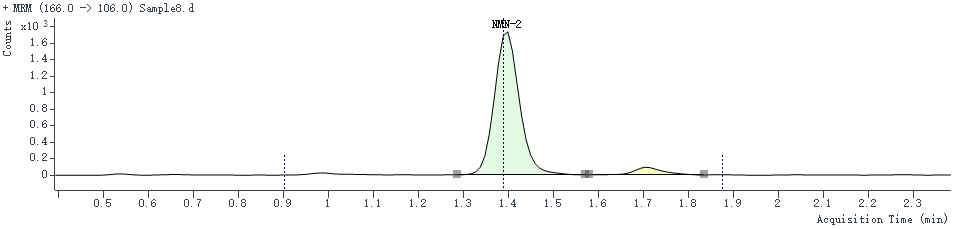

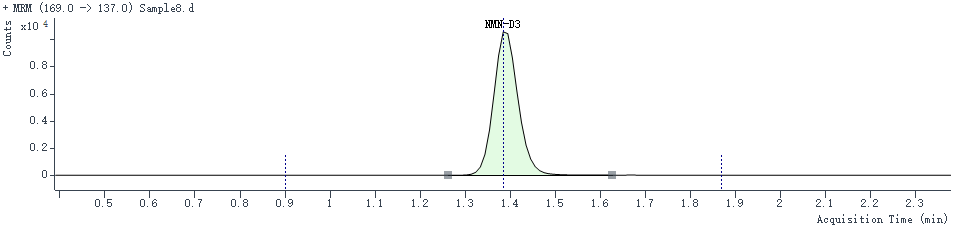

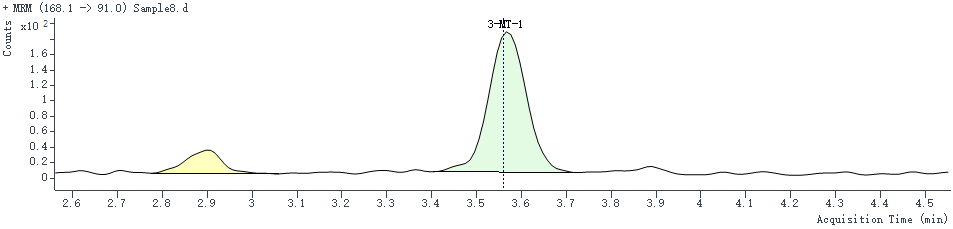

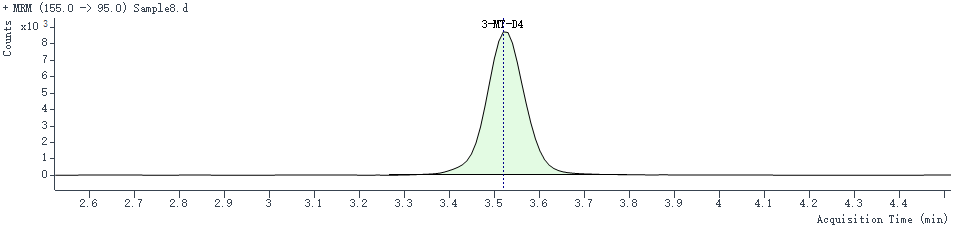

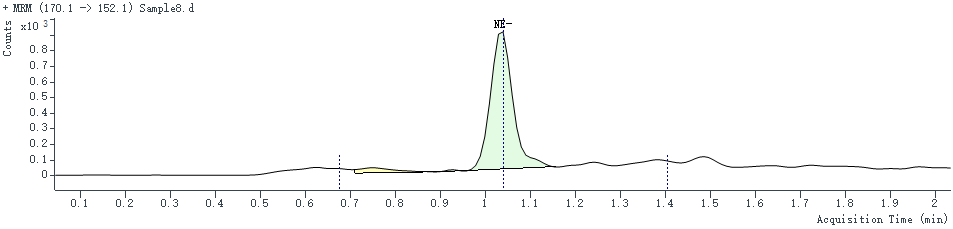

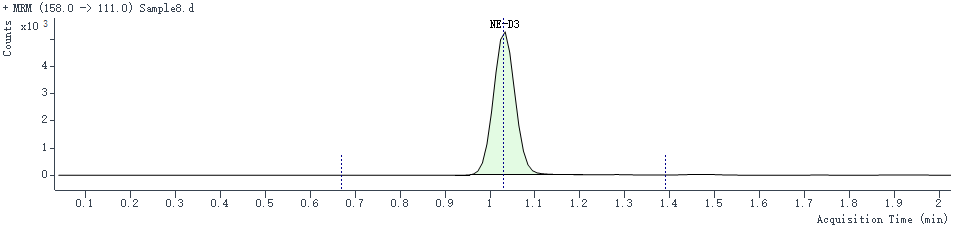

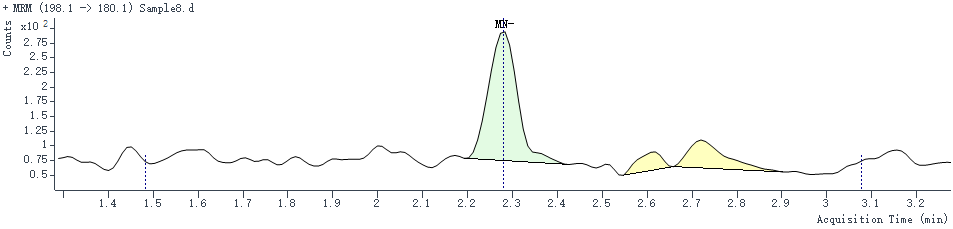

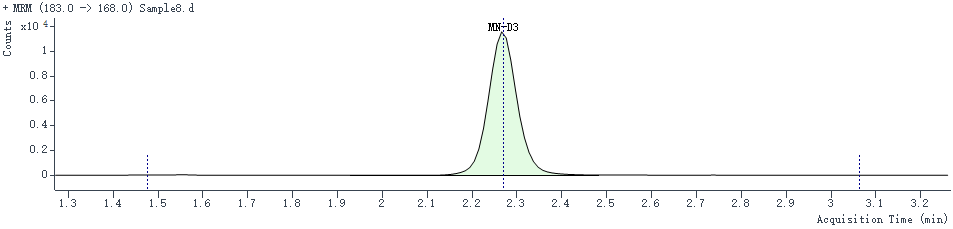


Table S1. Comparison of different methods for the detection of catecholamines.

| Methods | Sensitivity | Disadvantages | Advantages | Clinical application |
| --- | --- | --- | --- | --- |
| Immunoassays | ng/mL | Unable to detect catecholamine metabolites | Low instrument cost and easy to operate | Not widely used |
| Radioimmunoassay | ng/mL | Lower automation, higher cost, requires special precautions and licensing | Sensitive and specific, requiring specialized equipment | Not widely used |
| High-performance liquid chromatography (HPLC) | ng/mL | Not able to separate all compounds well | Easier to operate than mass spectrometry | Not widely used |
| Liquid chromatography-tandem mass spectrometry (LC-MS/MS) | pg/mL | High operational requirements | High throughput, high specificity, accurate results, high sensitivity | Widely used |

Table S2. Isotopic purities of the internal standards.

|  | Chemical Purity Specification | Isotopic Purity |
| --- | --- | --- |
| DA-D4 | 99.5% | ＞98% |
| E-D6 | 98.2% | 98.8% |
| NE-D3 | 95.8% | 98.6% |
| MN-D3 | 98.7% | 98.7% |
| NMN-D3 | 98.1% | 98.5% |
| 3-MT-D4 | ≥98% | 97.8% |

The molecular formulae of DA-*d*_4_, E-*d*_6_, NE-*d*_3_, MN-*d*_3_, NMN-*d*_3_, and 3-MT-*d*_4_ were C_8_H_8_ClD_4_NO_2_, C_9_H_7_D_6_NO_3_, C_9_H_11_ClD_3_NO_3_, C_10_H_13_D_3_ClNO_3_, C_9_H_11_D_3_ClNO_3_, and C_9_H_9_D_4_NO_2_.HCl, respectively.

Abbreviations: DA, dopamine; E, epinephrine; NE, norepinephrine; MN, metanephrine; NMN, normetanephrine; and 3-MT, 3-methoxytyramine.

Table S3. Compositions of calibration standards and quality controls (concentrations in picograms per milliliter).

| Substance | DA | E | NE | MN | NMN | 3-MT |
| --- | --- | --- | --- | --- | --- | --- |
| Working standard sample 1 | 4.5 | 2.5 | 4.5 | 2.5 | 2 | 0.3 |
| Working standard sample 2 | 45 | 25 | 45 | 25 | 20 | 3 |
| Working standard sample 3 | 225 | 125 | 225 | 125 | 100 | 15 |
| Working standard sample 4 | 900 | 500 | 900 | 500 | 400 | 60 |
| Working standard sample 5 | 1800 | 1000 | 1800 | 1000 | 800 | 120 |
| Working standard sample 6 | 3600 | 2000 | 3600 | 2000 | 1600 | 240 |
| Low value quality control | 90 | 50 | 90 | 50 | 40 | 6 |
| Middle value quality control | 450 | 250 | 450 | 250 | 200 | 30 |
| High value quality control | 2880 | 1600 | 2880 | 1600 | 1280 | 192 |

Abbreviations: DA, dopamine; E, epinephrine; NE, norepinephrine; MN, metanephrine; NMN, normetanephrine; and 3-MT, 3-methoxytyramine.

Table S4. Elution conditions for the separation of DA, E, NE, MN, NMN, and 3-MT by LC-MS/MS at a flow rate of 0.50 mL/min.

|  | Time (min) | A (%) | B (%) |
| --- | --- | --- | --- |
| 1 | 0.00 min | 100.0 % | 0.0 % |
| 2 | 3.00 min | 100.0 % | 0.0 % |
| 3 | 4.00 min | 15.0 % | 85.0 % |
| 4 | 5.00 min | 5.0 % | 95.0 % |
| 5 | 6.00 min | 5.0 % | 95.0 % |
| 6 | 6.10 min | 100.0 % | 0.0 % |
| 7 | 6.50 min | 100.0 % | 0.0 % |

Table S5. Mass spectrometry parameters for multiple reaction monitoring transitions of DA, E, NE, MN, NMN, and 3-MT in plasma and CSF samples by LC-MS/MS.

| No. | Analyte | Mode | Quantifier mass transition (m/z) | Fragmentor(V) | Collision energy (V) |
| --- | --- | --- | --- | --- | --- |
| 1 | DA | ESI+ | 154.1 > 91.0 | 80 | 26 |
| 2 | DA-IS | ESI+ | 158.1 > 140.9 | 70 | 8 |
| 3 | E | ESI+ | 166.1 > 77.0 | 111 | 41 |
| 4 | E-IS | ESI+ | 190.1 > 172.2 | 40 | 15 |
| 5 | NE | ESI+ | 170.1 > 152.1 | 80 | 2 |
| 6 | NE-IS | ESI+ | 158.0 > 111.0 | 130 | 20 |
| 7 | MN | ESI+ | 198.1 > 180.1 | 80 | 6 |
| 8 | MN-IS | ESI+ | 183.0 > 168.0 | 135 | 16 |
| 9 | NMN | ESI+ | 166.0 > 106.0 | 120 | 16 |
| 10 | NMN-IS | ESI+ | 169.0 > 137.0 | 120 | 16 |
| 11 | 3-MT | ESI+ | 168.1 > 91.0 | 80 | 26 |
| 12 | 3-MT-IS | ESI+ | 155.0 > 95.0 | 135 | 20 |

Abbreviations: DA, dopamine; E, epinephrine; NE, norepinephrine; MN, metanephrine; NMN, normetanephrine; 3-MT, 3-methoxytyramine; IS, internal standard; and ESI, electrospray ionization.

Table S6. Calibration linearity of DA, E, NE, MN, NMN, and 3-MT solutions prepared with the dilution method for sample pretreatment.

| Analyte | DA | | E | | NE | | MN | | NMN | | 3-MT | |
| --- | --- | --- | --- | --- | --- | --- | --- | --- | --- | --- | --- | --- |
|  | Exp. Conc. | Final Conc. | Exp. Conc. | Final Conc. | Exp. Conc. | Final Conc. | Exp. Conc. | Final Conc. | Exp. Conc. | Final Conc. | Exp. Conc. | Exp. Conc. |
| S1.d | 4.5 | 1444.31 | 2.5 | 2.50 | 4.5 | 4.50 | 2.5 | 2.51 | 2 | 2.01 | 0.3 | 0.32 |
| S2.d | 45 | 159.12 | 25 | 16.30 | 45 | 70.53 | 25 | 24.09 | 20 | 3.93 | 3 | 44.77 |
| S3.d | 225 | 1558.32 | 125 | 119.92 | 225 | 240.77 | 125 | 126.84 | 100 | 87.38 | 15 | 0.47 |
| S4.d | 900 | 1824.03 | 500 | 480.03 | 900 | 824.19 | 500 | 492.70 | 400 | 369.03 | 60 | 54.31 |
| S5.d | 1800 | 2479.60 | 1000 | 980.84 | 1800 | 1818.86 | 1000 | 1001.94 | 800 | 866.37 | 120 | 117.51 |
| S6.d | 3600 | 3328.97 | 2000 | 2197.55 | 3600 | 3616.87 | 2000 | 2062.17 | 1600 | 1788.94 | 240 | 248.16 |

Abbreviations: Exp. Conc., expected concentration; Final Conc., final concentration; S, working standard sample.

Table S7. Dilution consistency for sample 6 diluted 10-fold, 5-fold, and 3-fold with 1% low-fatty acid bovine serum albumin (BSA) with five replicates for each dilution. The dilution consistency was considered acceptable if the recovery was between 85% and 115% and coefficient of variation (CV) was within ± 15%.

| Analyte | **Plasma** | | | | **CSF** | | | |
| --- | --- | --- | --- | --- | --- | --- | --- | --- |
|  | Theoretical concentration (pg/mL)/ Dilution ratio | Calculation concentration | Recovery (%) | CV (%) (n=5) | Theoretical concentration (pg/mL)/ Dilution ratio | Calculation concentration | Recovery (%) | CV (%) (n=5) |
| DA | 9000/3 | 2851.92 | 95.06 | 4.94 | 9000/3 | 2914.98 | 97.17 | 2.83 |
| E | 4500/3 | 1602.30 | 106.82 | 6.82 | 4500/3 | 1636.43 | 109.10 | 9.10 |
| NE | 9000/3 | 3041.03 | 101.37 | 1.37 | 9000/3 | 3064.70 | 102.16 | 2.16 |
| MN | 4500/3 | 1685.06 | 112.34 | 12.34 | 3000/3 | 1131.20 | 113.12 | 13.12 |
| NMN | 4500/3 | 1360.06 | 90.67 | 9.33 | 4500/3 | 1344.68 | 89.65 | 10.35 |
| 3-MT | 600/3 | 208.46 | 104.23 | 4.23 | 600/3 | 210.78 | 105.39 | 5.39 |
| DA | 10000/5 | 1824.22 | 91.21 | 8.79 | 10000/5 | 1832.41 | 91.62 | 8.38 |
| E | 5000/5 | 1036.80 | 103.68 | 3.68 | 5000/5 | 1048.01 | 104.80 | 4.80 |
| NE | 10000/5 | 1943.59 | 97.18 | 2.82 | 10000/5 | 1995.73 | 99.79 | 0.21 |
| MN | 5000/5 | 1072.44 | 107.24 | 7.24 | 10000/5 | 1853.46 | 92.67 | 7.33 |
| NMN | 5000/5 | 859.34 | 85.93 | 14.07 | 5000/5 | 907.78 | 90.78 | 9.22 |
| 3-MT | 600/5 | 131.82 | 109.85 | 9.85 | 600/5 | 137.74 | 114.79 | 14.79 |
| DA | 30000/10 | 2962.22 | 98.74 | 1.26 | 30000 | 2986.51 | 99.55 | 0.45 |
| E | 15000/10 | 1688.19 | 112.55 | 12.55 | 15000 | 1674.98 | 111.67 | 11.67 |
| NE | 30000/10 | 3064.70 | 102.16 | 2.16 | 30000 | 3224.67 | 107.49 | 7.49 |
| MN | 15000/10 | 1711.75 | 114.12 | 14.12 | 15000 | 1476.12 | 98.41 | 1.59 |
| NMN | 15000/10 | 1427.74 | 95.18 | 4.82 | 15000 | 1436.03 | 95.74 | 4.26 |
| 3-MT | 2000/10 | 217.07 | 108.53 | 8.53 | 2000 | 219.48 | 109.74 | 9.74 |

Abbreviations: CSF, cerebrospinal fluid; CV, coefficient of variation; DA, dopamine; E, epinephrine; NE, norepinephrine; MN, metanephrine; NMN, normetanephrine; and 3-MT, 3-methoxytyramine.

Table S8. Stability of the plasma samples at LQC, MQC, HQC concentrations were validated with all the deviations within 15% under different conditions.

| Analyte | | Mean (pg/mL) | | | CV (%) |
| --- | --- | --- | --- | --- | --- |
|  |  | room temperature for 2 h (n=5) | -20◦C for 3 days (n=5) | -80◦C for 7 days (n=5) |  |
| DA | LQC | 107.01 | 107.14 | 108.35 | 1.98 |
|  | MQC | 98.20 | 97.33 | 103.03 | 4.17 |
|  | HQC | 101.43 | 102.47 | 102.28 | 1.93 |
| E | LQC | 111.16 | 107.00 | 110.68 | 2.78 |
|  | MQC | 99.25 | 99.02 | 110.75 | 6.06 |
|  | HQC | 110.38 | 108.39 | 105.88 | 2.55 |
| NE | LQC | 104.93 | 107.32 | 104.60 | 2.81 |
|  | MQC | 107.67 | 98.32 | 103.99 | 5.5 |
|  | HQC | 105.43 | 105.02 | 105.96 | 1.75 |
| MN | LQC | 106.52 | 109.08 | 109.19 | 2.57 |
|  | MQC | 96.1 | 98.76 | 105.55 | 5.52 |
|  | HQC | 104.47 | 106.84 | 106.92 | 1.97 |
| NMN | LQC | 100.80 | 104.90 | 100.14 | 4.45 |
|  | MQC | 101.48 | 102.26 | 105.78 | 5.47 |
|  | HQC | 101.23 | 102.20 | 103.49 | 2.23 |
| 3-MT | LQC | 102.85 | 104.95 | 109.00 | 4.44 |
|  | MQC | 97.78 | 97.86 | 104.90 | 4.95 |
|  | HQC | 105.40 | 110.00 | 104.58 | 4.66 |

Abbreviations: CV, coefficient of variation; DA, dopamine; E, epinephrine; NE, norepinephrine; MN, metanephrine; NMN, normetanephrine; 3-MT, 3-methoxytyramine; LQC, low-value quality control; MQC, middle-value quality control; and HQC, high-value quality control.

Table S9. The stability of cerebrospinal fluid samples. The stability of LQC, MQC, and HQC samples were validated with all the deviations within 15% under different conditions.

| Analyte | | Mean (pg/mL) | | | CV (%) |
| --- | --- | --- | --- | --- | --- |
|  |  | room temperature for 2 h (n=5) | -20◦C for 3 days (n=5) | -80◦C for 7 days (n=5) |  |
| DA | LQC | 106.65 | 105.93 | 98.85 | 4.36 |
|  | MQC | 100.45 | 98.3 | 98.71 | 3.3 |
|  | HQC | 98.54 | 98.00 | 98.89 | 1.58 |
| E | LQC | 110.31 | 107.72 | 102.60 | 4.52 |
|  | MQC | 99.91 | 99.9 | 98.46 | 2.85 |
|  | HQC | 103.1 | 102.88 | 102.01 | 2.4 |
| NE | LQC | 104.26 | 105.42 | 96.97 | 5.02 |
|  | MQC | 101.27 | 105.05 | 107.48 | 6.13 |
|  | HQC | 98.83 | 98.97 | 98.48 | 2.43 |
| MN | LQC | 101.58 | 103.03 | 93.69 | 4.7 |
|  | MQC | 97.93 | 99.01 | 99.68 | 3.64 |
|  | HQC | 97.58 | 98.52 | 99.04 | 2.07 |
| NMN | LQC | 101.75 | 99.88 | 97.75 | 4.84 |
|  | MQC | 102.23 | 103.07 | 104.32 | 3.89 |
|  | HQC | 98.85 | 100.04 | 99.63 | 1.78 |
| 3-MT | LQC | 103.55 | 102.46 | 95.10 | 4.93 |
|  | MQC | 99.77 | 98.41 | 99.34 | 2.89 |
|  | HQC | 103.15 | 103.04 | 103.38 | 4.24 |

Abbreviations: CV, coefficient of variation; DA, dopamine; E, epinephrine; NE, norepinephrine; MN, metanephrine; NMN, normetanephrine; 3-MT, 3-methoxytyramine; LQC, low-value quality control; MQC, middle-value quality control; and HQC, high-value quality control.
